# Root and canal morphology of third molar teeth

**DOI:** 10.1038/s41598-023-34134-7

**Published:** 2023-04-27

**Authors:** Aladdin Ahmad Al-Qudah, Hamzeh A. Barakat Bani Younis, Lama Adel Awawdeh, Alaa Daud

**Affiliations:** 1 Department of Conservative Dentistry, Jordan University of Science and Technology, P.O. Box 3030, Irbid, 22110 Jordan; 2 College of Dental Medicine, QU Health, Qatar University, Doha, Qatar

**Keywords:** Anatomy, Oral anatomy

## Abstract

Restorative and prosthetic considerations usually necessitates endodontic treatment of third molars in order to retain them as a functional component of the dental arch. However, the anatomy of third molars has been described as unpredictable. To date, there has been little published work on root and canal morphology of third molars, with an adequate sample size. The aim of this study was to investigate root and canal morphology of third molars. Maxillary and mandibular third molars were gathered from dental surgeries within north Jordan. Following access cavity preparation, pulp tissue was removed and root canals stained. Teeth were subject to examination after rendering them clear by immersion in methyl salicylate, and the following features evaluated: (1) number of roots; (2) number and type of root canals; (3) number and position of lateral canals; and (4) presence of inter-canal communications. Out of the examined 592 maxillary third molars, 69.9% had three roots, 10.81% had one, 9.79% had two, 9.12% had four, and 0.34% had five roots. Most had three (52.36%) and four canals (28.2%) with less frequency of two (11.48%), one (5.91%) and five canals (2.03%). Of the 639 mandibular third molars, 89.76% had two roots, 7.35% had three, 2.5% had one, and 0.47% had four roots. Most had three (55.71%) and two canals (29.42%) with less frequency of four (13.61%), one (1.09%) and five canals (0.15%). The majority of maxillary third molars had 3 roots, while the majority of mandibular third molars had two. Overall, nearly half of the maxillary and mandibular third molars had three canals. New canal configurations, not previously described in the literature nor included in Vertucci’s classifications, were identified in both maxillary and mandibular third molars.

## Introduction

A general tendency towards retaining teeth rather than extraction is evident nowadays as part of contemporary dental practice. Accordingly, it is more common to endodontically treat third molars, especially if they are to serve as appropriate abutments for fixed prosthesis^1^.

Locating and negotiating all root canals prior to chemo-mechanical debridement is a prerequisite to the success of root canal therapy^2^. Incomplete debridement and obturation of the canal space may result from false assumptions about root and canal morphology, leading to endodontic failure. Studies have shown that a common cause of failure of root canal treatment is inadequate canal cleaning and obturation due to untreated or missed canals^3,4^.

Anatomical variations and unusual root patterns require an operator with a clear knowledge of root canal morphology. A wide range of investigative techniques have been carried out in undergraduate and postgraduate level, in vitro and in vivo, 2-dimensional and 3-dimentional, in order to estimate, analyze and teach root canal morphology. These techniques include scanning electron microscopy, tooth demineralisation^5–7^, cross-sectional inspections^8^, classic and digital radiographic techniques^9^, cone beam computed tomography (CBCT)^9,10^, 3- dimensional micro-computed tomography (micro-CT)^2^, 3D-printing of teeth with complex anatomy from CBCT’s^11^ and virtual reality (haptic) simulation^12^.

Many authors have previously explored the anatomy of root canals, however, not many have included third molars. Pineda and Kuttler^13^ using radiographic techniques studied the mesiobuccal (MB) root of maxillary third molars and the mesial and distal roots of mandibular third molar. Green^8^ limited his study to the MB root of maxillary third molars and the mesial root of mandibular third molars, detailing the number of major canals and the number of apical foramina. Sidow and coworkers^7^ provided morphological descriptions of third molars in USA. Ng and colleagues^14^ studied maxillary molars, including third molars, in Burmese population. Numerous studies reported that root canal systems may vary depending on race and geographic area^6,15^.

Representation of commonly occurring root canal systems are dependent largely on studies carried out in Europe and North America, and relate to teeth of mainly Caucasoid origin. Nonetheless, these descriptions may not be fully applicable to teeth of non-Caucasoid origin. A study investigating the root and canal morphology of human third molars in a Jordanian subpopulation showed a range of morphologies, with the number of roots and root canals not affected by patient’s gender, age or tooth location^16^. However, the sample size did not exceed 159 teeth (89 maxillary and 70 mandibular). Given the known variations and complexity of root canal anatomy of third molar teeth, such a small sample size may not give accurate descriptions of root canal systems for these teeth. Therefore, the aim of this study was to examine the root and canal morphology of maxillary and mandibular third molars utilizing a large sample size (1231 teeth) from a Jordanian population, using canal staining and root clearing technique.

## Materials and methods

Sample teeth, extracted from Jordanian patients, were collected from various dental clinics within north Jordan.

Dentists were informed that teeth were to be used for research purposes unidentified. The gender and age of patients, and reason for extraction were not recorded. This study was approved by the Research Ethical committee at Jordan University of Science & Technology. Informed consent was obtained from all subjects and/or their legal guardian(s); in accordance with the guidelines governing the practice of dentistry in Jordan. All methods were carried out in accordance with relevant guidelines and regulations. Five hundred and ninety-two (n = 592) extracted maxillary third molars and six hundred and thirty-nine (n = 639) mandibular third molars were identified/confirmed on the basis of crown morphology by two independent examiners according to accepted criteria^17^. A consensus was reached by examiners to minimize error. Teeth with premature roots or fractured roots were excluded.

Teeth were initially stored in a 10% formalin solution until the investigation started. They were then immersed in 3.25% sodium hypochlorite solution (Hypex Bleach; Jordan Chemical Industries Co., Amman, Jordan) for two hours. Subsequently, remaining soft tissue and calculus were removed by scaling. Cleaned teeth were then examined visually to record their morphology and number of roots.

Calibrated clinicians prepared access cavities, followed by removal of pulp tissues through immersion in 3.25% sodium hypochlorite overnight, prior to placing in an ultrasonic bath for thirty minutes. Teeth were left to dry over night after rinsing under running tap water for 2 h. Indian ink (Sanford rotring GmbH, Hamburg, Germany) was injected into the canal system utilizing an endodontic irrigation syringe with a gauge 27 needle (BU Kwang Medical Inc., Seoul, Korea)^18^ To spread ink through the canal system, the root apex was immediately placed over small vacuum holes, linked to a central suction system until ink exited the apical foramina^18^.

A 10% nitric acid solution was used to demineralize the teeth for 5–7 days at room temperature (21 °C). Demineralization was checked by a dental probe that penetrated the tooth upon gentle pressure. The acid solution was renewed daily. Teeth were dried and dehydrated using an ascending concentrations of ethyl alcohol (70%, 95% and 99%) for 12 h each after washing for 4 h under running tap water. Finally, the dehydrated teeth were rendered transparent by immersing in methyl salicylate for around 2 h^18^and were stored in this solution until they were examined. The transparent specimens were examined by the naked eye under a halogen light and the following observations were made:

(1) Number of roots; (2) Number and type of root canals (defined as the largest number of canals visualized); (3) Frequency of C-shaped canals; (4) Number and position of lateral canals, and (5) Number of inter-canal communication.

Teeth were observed by two calibrated and experienced examiners; an endodontist and an endodontic resident, and this proved to be a straightforward process. A consensus was reached by examiners to minimize error.

**Figure 1:**
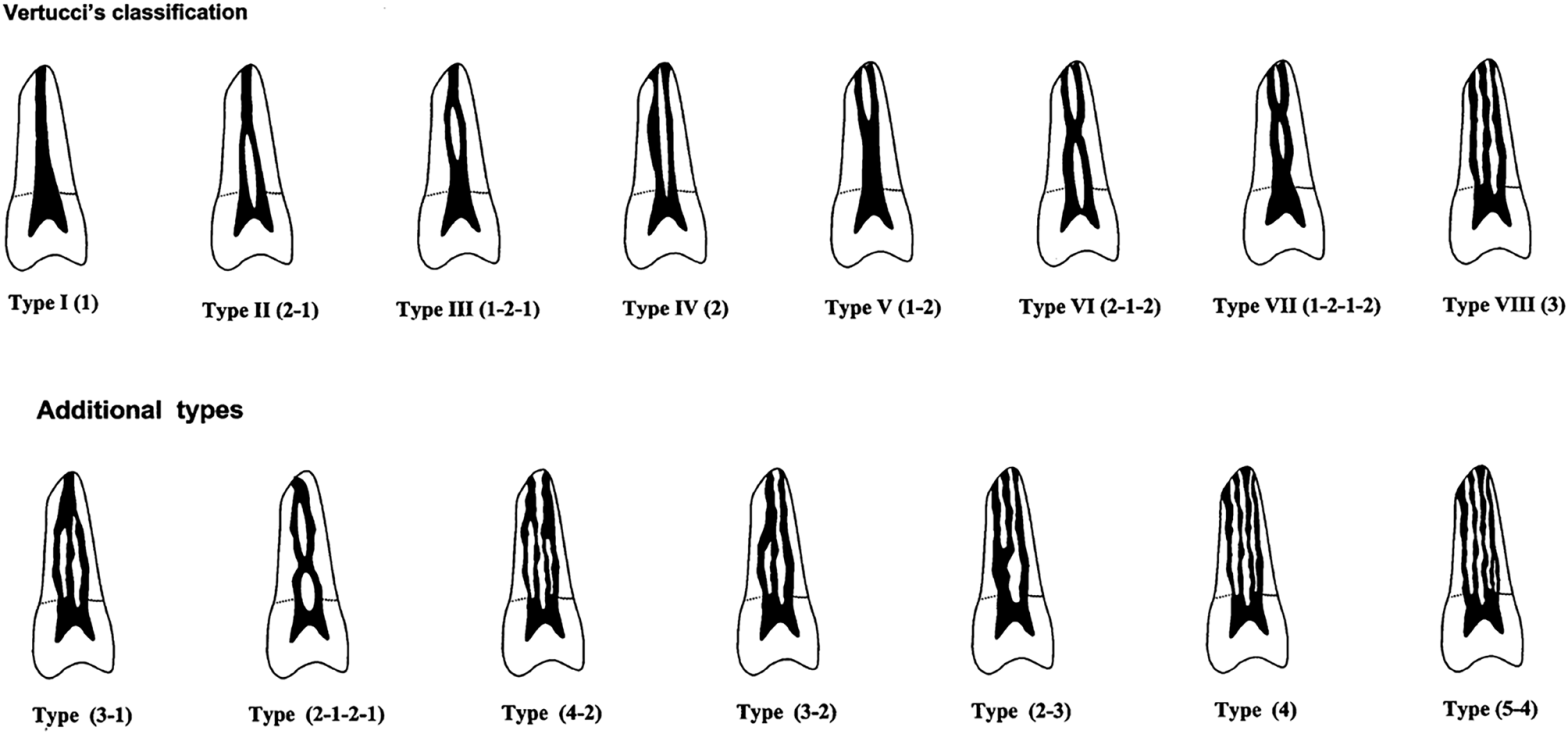
Classification of canal system by Vertucci^19^, and the additional modifications^20^.

Canal configurations were grouped into the eight types of Vertucci’s classification^19^ with additional modifications by Gulabivala et al.^20^, as follows (Fig. 1):

- *Type I* A single canal with one foramen.
- *Type II* Two separate canals, joining at the apical third to form one canal.
- *Type III* One canal leaving the pulp chamber, dividing into two that subsequently reunite and exit as one canal.
- *Type IV* Two separate canals all the way to the apex.
- *Type V* One canal that divides just short of the apex into two separate canals with separate foramina.
- *Type VI* Two canals that unite in the root and then divide again at the apex.
- *Type VII* One canal that divides, reunites, and finally: exits through two apical foramina.
- *Type VIII* Three separate canals in one root.

## Results

### Number of roots and their morphology

Data for the root morphology of the examined teeth are presented in Table 1. Most (69.9%) of maxillary third molars had three roots, whereby the majority (41%) had separate roots, followed by teeth with all-fused roots (21%). Lower prevalence (10.8%) of single rooted teeth was observed with the majority being conical-shaped (9.1%) and the rest (1.7%) being C-shaped. The two-root variant was observed in 9.8% of teeth, which were either separate (5%) or fused (5%). Four and five roots were present in 9.1% and 0.34% of teeth, respectively. Five rooted teeth were the least prevalent and were recorded for the first time. They had three buccal fused roots and two lingual separate roots.

**Table 1:** Classification of maxillary and mandibular third molars according to root number and morphology.

No. of roots	Maxillary molars			Mandibular molars		
	No. of teeth and (%)	Root shape and number (%)		No. of teeth and (%)	Root shape and number (%)	
1 root	64 (10.8%)	- Conical	54, (9.1%)	16 (2.5%)	- Conical	4, (0.63%)
		- C-shaped	10, (1.7%)		- C-shaped	12, (1.87%)
2roots	58 (9.8%)	- Separate	27, (4.6%)	573 (89.7%)	- Separate	510, (79.8%)
		- Fused	31, (5.2%)		- Fused	63, (9.9%)
3 roots	414 (69.9%)	- Separate	243, (41.0%)	47 (7.4%)	- Separate	18, (2.81%)
		- B roots fused	23, (3.9%)		- M & DL fused	18, (2.81%
		- MB & P fused	23, (3.9%)		- D & DL fused	
		- All fused	125, (21.1%)		- All fused	2, (0.31%)
						9, (1.41%)
4 roots	54 (9.1%)	- All fused	26, (4.4%)	3 (0.47%)	–	3, (0.47%)
		- All separate	6, (1.0%)			
		- MB & MP fused	10, (1.7%)			
		- Palatal roots fused	1, (0.17%)			
		- DB & Ps fused	1, (0.17%)			
		- Bs fused & Ps roots fused	1, (0.17%)			
		- Bs roots fused				
		- MB & DB & MP fused	2, (0.34%)			
			7, (1.2%)			
5 roots	2 (0.34%)	–	2, (0.34%)	–		

Of the 639 mandibular third molars, there was high prevalence (89.7%) of two roots; with nearly 80% being separate while the rest fused. Lower prevalence of three (7.4%), single (2.5%) and four roots (0.47%) was observed.

### Number and type of canal system

**Figure 2:**
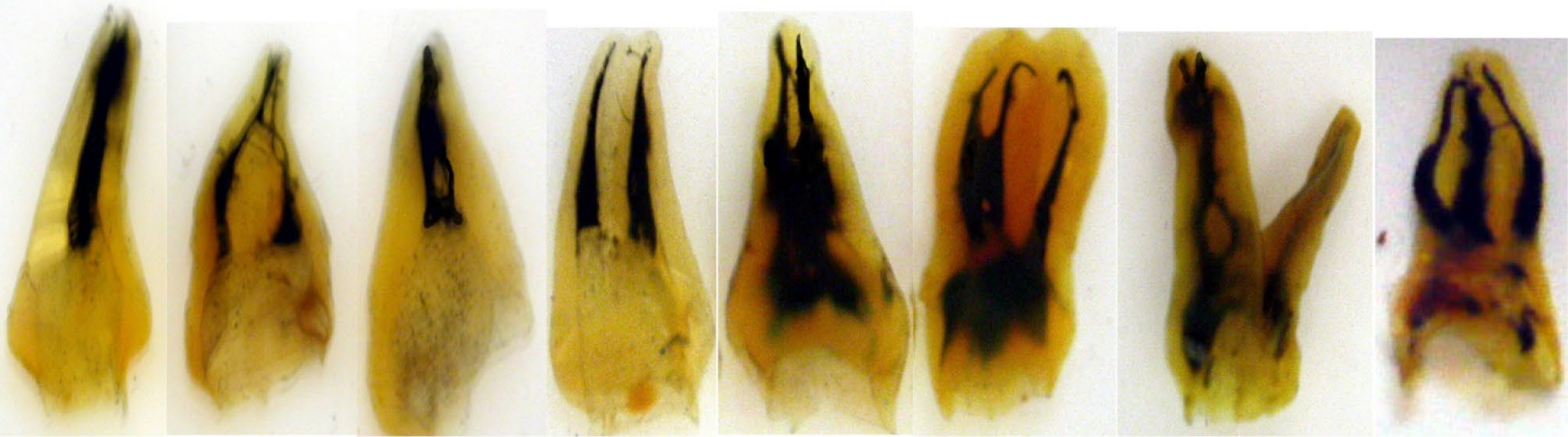
The eight types of Vertucci’s canal configuration in cleared maxillary third molars.

**Figure 3:**
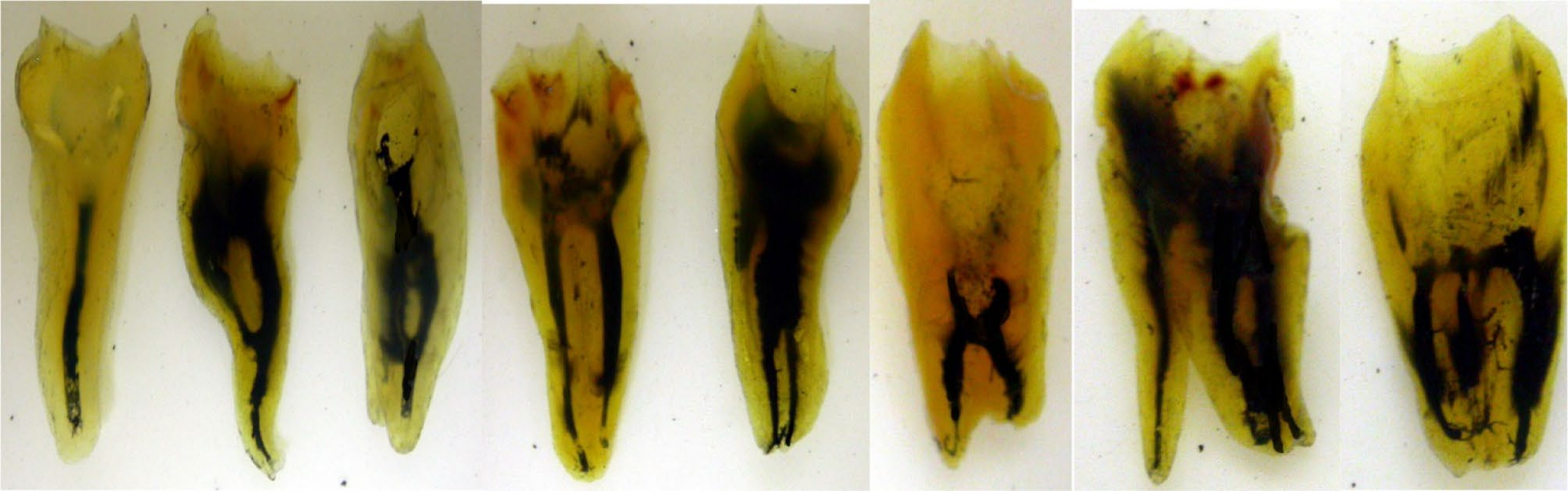
The eight types of Vertucci’s canal configuration in cleared mandibular third molars.

The data for canal numbers and configuration are provided in Tables 2, 3 and 4. The eight types of Vertucci’s canal configuration were found in both maxillary and mandibular third molars (Figs. 2, 3). New canal configurations, not included in Vertucci’s classifications, were found in both maxillary and mandibular third molars: these were type (1-3, 1-3-1, 3-1, 3-2, 2-3, 3-4, 4-1, 4-2, 4-3, 4-4, 4-5) (Fig. 3).

**Table 2:** Number and percentage of canals per toot.

Type of molar	Number of teeth	Number and percentage of canals per tooth				
		1	2	3	4	5
Maxillary third molars	592	35	68	310	167	12
		5.91%	11.48%	52.36%	28.2%	2.03%
Mandibular third molars	639	7	188	356	87	1
		1.09%	29.42%	55.71%	13.61%	0.15%

**Table 3:** Number and percentage of the common canal system types in maxillary third molars.

Group	No. of roots	Type I	Type II	Type III	Type IV	Type V	Type VI	Type VII	Type VIII	Additional types										
		1	2-1	1-2-1	2-2	1-2	2-1-2	1-2-1-2	3-3	1-3-1	3-1	3-2	3-4	1-3	2-3	4-1	4-2	4-3	4-4	4-5
Three separate roots:																				
MB root	243	153 62.96	16 6.58%	9 3.7%	29 11.93%	23 9.46%	7 2.88%	5 2.05%	1 0.41%	–	–	–	–	–	–	–	–	–	–	–
DB root	243	243 100%	–	–	–	–	–	–	–	–	–	–	–	–	–	–	–	–	–	–
P root	243	242 99.59%	–	1 0.41	–	–	–	–	–	–	–	–	–	–	–	–	–	–	–	–
Three roots: B fused																				
MB & DB fused	23	–	6 26.08%	–	12 52.17%	1 4.35%	–	–	2 8.69%	–	–	–	–	–	2 8.69%	–	–	–	–	–
P	23	23 100%	–	–	–	–	–	–	–	–	–	–	–	–	–	–	–	–	–	–
Three roots																				
MB & P fused	23	–	–	–	13 56.52%	–	2 8.69%	–	3 13.04%	–	–	1 4.35%	2 8.69%	–	2 8.69%	–	–	–	–	–
DB	23	23 100%	–	–	–	–	–	–	–	–	–	–	–	–	–	–	–	–	–	–
Three roots all fused	125	–	–	–	–	–	–	–	80 64.0%	–	15 12.0%	12 9.6%	10 8.0%	–	3 2.4%	–	–	1 0.8%	4 3.2%	–
Two roots: separate																				
Buccal	27 77.77%	21 7.4%	2 14.8%	–	–	4	–	–	–	–	–	–	–	–	–	–	–	–	–	–
Palatal	27	27 100%	–	–	–	–	–	–	–	–	–	–	–	–	–	–	–	–	–	–
Two roots: fused	31	–	1 3.2%	–	23 74.19%	–	2 6.45%	–	–	–	–	2 6.45%	–	–	2 6.45%	–	–	1 3.22%	–	–

**Table 4:** Number and percentage of the common canal system types in mandibular third molars.

Group	No. of Roots	Type I	Type II	Type III	Type IV	Type V	Type VI	Type VII	Type VII	Additional types								
		1	2-1	1-2-1	2-2	1-2	2-1-2	1-2-1-2	3-3	1-3-1	3-1	3-2	3-4	2-3	4-1	4-2	4-3	4-4
Three separate roots																		
M root	18	13 72.22%	1 5.55%	–	1 5.55%	3 16.66%	–	–	–	–	–	–	–	–	–	–	–	–
D root	18	16 88.88%	–	–	–	2 11.11%	–	–	–	–	–	–	–	–	–	–	–	–
L root	18	18 100%	–	–	–	–	–	–	–	–	–	–	–	–	–	–	–	–
Three roots:																		
M&DL fused 83.33%	18	–	–	–	15 5.55%	1 5.55%	–	–	1 5.55%	–	–	–	–	1 5.55%	–	–	–	–
D root	18	16 88.88%	1 5.55%	–	1 5.55%	–	–	–	–	–	–	–	–	–	–	–	–	–
Three roots: D fused																		
D&DL fused	2	–	–	–	1 50%	1 50%	–	–	–	–	–	–	–	–	–	–	–	–
M	2	1 50%	1 50%	–	–	–	–	–	–	–	–	–	–	–	–	–	–	–
Three roots all fused	9	–	–	–	–	–	–	–	4 44.44%	–	–	1 11.11%	4 44.44%	–	–	–	–	–
Two roots: separate																		
M	510	140 27.45%	97 19.01%	15 2.94%	235 46.08	15 2.94%	15 0.78%	4 0.78%	4 0.78%	–	–	–	–	–	–	–	–	–
D	510	455 89.21%	4 0.78%	6 1.17%	42 8.23%	3 0.58%	–	–	–	–	–	–	–	–	–	–	–	–
Two roots: fused	63	–	8 12.69%	–	30 47.62%	1 1.58%	–	–	4 6.35%	–	6 9.52%	5 7.93%	1 1.58%	4 6.35%	1 1.58%	1 1.58%	–	3 4.76%

#### Maxillary third molars

The majority of maxillary third molars had three canals (52.4%) followed by four (28.2%) with lower prevalence of two (11.5%), one (5.9%) and five canals (2.0%) (Table 2). In the teeth with conical roots, type I was the most prevalent canal configuration (64.81%), and the rest were distributed amongst other types. The C-shaped root showed wide variation of canal number and types. In the teeth with two separate roots, type I was the most common canal configuration in the buccal root (77.77%), followed by type V (14.8%), and type II (7.4%); all the palatal roots had type I (100%). The teeth with two fused roots had high prevalence of type IV canal configuration (74.19%) and the rest were distributed amongst several additional types.

In the teeth with three separate roots, the prevalence of two canals in MB root was (36.6%) in the form of type II (6.58%), type III (3.7%), type IV (11.93%), type V (9.46%), type VI (2.88%) and type VII (2.05%) configurations. While the percentage of one canal was (62.96%). All the DB roots (100%) and (99.59%) of palatal roots had type I canal configuration (Table 3).

Teeth with three fused roots showed high variety of canal configurations. In teeth with fused MB & DB roots the most common configuration was type IV (52.17%); the remainders were distributed amongst the other types including the additional ones. All palatal roots had type I (100%) canal configuration. In the 3-rooted teeth with fused MB and P roots the most prevalent configuration was type IV (56.52%) the remainders were distributed amongst the other types including the additional ones. All the DB roots I (100%) had type I canal configuration. Teeth with all fused three roots had type VIII (64%), and the rest had additional configurations (36% collectively).

Teeth with four roots showed wide variation in canal morphology. In the teeth with separate roots, all the MB roots had one (type I, 83.33%), and two canals (type V, 16.66%), and all the other roots had type I canal configuration. The fused roots showed the highest variation in root morphology and canal configuration.

**Figure 4:**
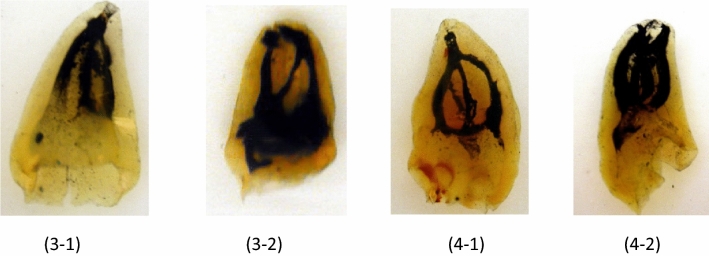
Cleared maxillary third molars with additional canal configurations.

All teeth with five roots had the additional type (2-3) in the buccal three-fused roots, and type I in the other roots. There was a high prevalence of lateral canals (11.77%) with the highest being in the apical third. Inter-canal communications were found in 12.48% of teeth (Table 3). New canal configurations, not included in Vertucci’s classifications, were found in maxillary third molars: these were type (3-1, 3-2, 4-1, 4-2) as in Fig. 4.

#### Mandibular third molars

The majority of teeth had three canals (55.71%), followed by two (29.42%), and four (13.61%) with lower prevalence of one (1.09%) and five canals (0.15%) (Table 2).

In the teeth with conical roots, type I was the most prevalent canal configuration (75%), and rest were type III (25%). The C-shaped roots showed type I, II, IV, V and VI (33.33%, 16.66%, 16.66%, 25%, and 8.33%). In the teeth with two separate roots, mesial root with two canals were found in 71.75% of the teeth. The most prevalent canal configuration in the mesial root was type IV (46.08%), followed by type I (27.45%), and type II (19.01%). The remainders were distributed amongst several other types. The distal root showed high prevalence (89.21%) of type I canal configuration while the remaining roots were distributed amongst several types. Teeth with fused two roots had a spread of canal configuration including type IV (47.62%) and type II (12.69%), while the rest were distributed amongst several additional types.

In the teeth with three separate roots, type I was present in all distolingual roots (100%), while the distal root had type I (88.88%) and type V (11.11%) canal configurations. The mesial root had type I (72.22%), type V (16.66%), and equal proportions of type II and IV configurations (5.55%). Teeth with three fused roots showed high variety of canal configurations. The teeth with all fused roots had type VIII (44.44%), and the rest had additional configurations (55.55% collectively). Most of the 3-rooted teeth with fused mesial and distolingual roots had type IV (83.33%), while most of distal root had type I configuration (88.88%). Teeth with fused distal and distolingual roots had equal proportions of type IV and type V configuration.

**Figure 5:**
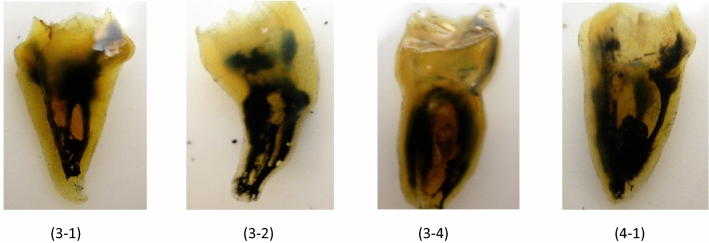
Cleared mandibular third molars with additional canal configurations.

Finally, teeth with four roots showed two forms: one form with fused distal roots, exhibiting type V configuration & separate mesial roots exhibiting type I configuration. The other form showed all separate roots demonstrating type I configuration in all (100%) of the ML, DL, and DB roots, while all (100%) MB roots showed type V canal configuration. The apical part of the root showed increasingly prevalent lateral canals, with the highest number at the apical third (10.04%). Inter-canal communications were found in 10.13% of teeth. C-shaped canals were found in 1.5% of teeth. New canal configurations, not included in Vertucci’s classifications, were found in mandibular third molars: (3-1, 3-2, 3-4, 4-1) as in Fig. 5.

## Discussion

Third molar teeth could be challenging for dentists to treat endodontically owing to their location within the jaw, rendering them difficult to access, in addition to their aberrant occlusal anatomy and unpredictable root morphology. Inevitably, pulpally-involved third molars are extracted and rarely considered for root canal therapy^21–23^. However, restorative considerations often require these teeth to be endodontically treated, to be retained in the dental arch. Accordingly, knowledge of the root canal morphology of these teeth is imperative for successful endodontic treatment. Operators must be aware of the complexity of the root canal system to be able to perform cleaning and shaping successfully.

A wide range of investigative methods, to analyze teeth morphology, have been used in *vitro* and in *vivo*, including 2- dimensional and 3-dimensional techniques^5–7,24–26^. Staining and clearing technique is an approved, well known method of studying root canal morphology. It can reveal wide anatomical variations in different teeth in addition to fine canal details such as accessory canals^6,19,27,28^. However, nowadays other techniques such as micro-CT and CBCT imaging are more widely used for studying root and canal anatomy in laboratory and clinical observational studies, respectively. Micro-CT is now considered as the gold standard for laboratory root canal morphology studies, as it provides high level of accuracy compared to other techniques. In addition, it enables wide range of qualitative and quantitative analysis including high-resolution 2D and 3D reconstructed images for different parts of the root canal system. Consequently, Micro-CT is the method of choice when comprehensive quantitative analyses of root canal system are required. However, it is an expensive and time-consuming diagnostic tool, which can add restrictions to the number of teeth included in a micro-CT morphology study^28^. It is worth emphasizing that the present study aimed to investigate the main anatomical features of root and canal configurations (number and type of canals) of third molars and the clearing technique is known to provide this information. Third molars are among the least investigated teeth and are known to have a variable root and canal anatomy. Therefore, large sample size was used to reach a consistent conclusion about the common canal configurations in these teeth. The materials used in the clearing technique (acid, ink, etc.) are readily available and not expensive. It also does not require sophisticated software/devices, making it the method of choice for projects with limited budget or limited access to micro-CT/CBCT. Nevertheless, the clearing method remains beneficial only as a teaching/research tool, with little or no clinical applicability.

In the present study canals were categorized using Vertucci’s classification, which has been the most commonly used classification and has been beneficial when categorizing many canal configurations. Categorization of canal configurations, using this system, proved to be simple and straightforward. It is understood that some canal configurations were “non-classifiable” in this system. Therefore, additional modifications by Gulabivala et al.^20^, were also used to include other types that are not covered by the Vertucci’s classification. Nevertheless, some new configurations have been reported in the present study for both maxillary and mandibular third molars; namely type (3-1, 3-2, 4-1, 4-2) and type (3-1, 3-2, 3-4, 4-1) respectively. These are readily explained by the numbers that described such types (e.g. type 3-1 indicates three separate canals joining to exit in one foramen, and so on). The classification system used in the present study gives a clear conclusion about the number and type of canals in the investigated teeth, which is consistent with the aim of the study.

In recent years new coding system for classifying root and canal morphology, accessory canals and anomalies has been introduced^29,30^. This system of classification is increasing in popularity and the authors of the present manuscript will consider using it in future investigations using CBCT and micro CT.

Many authors have investigated the anatomy of root canals, but only a few studies have encompassed third molar teeth. Kuzekanani and co-workers^31^, investigated root morphology of maxillary and mandibular third molars in an Iranian sub-population. In 2017, Khawaja et al.^4^ investigated root canal morphology of mandibular third molars by conventional endodontic treatment. Tomaszewska et al.^26^, used micro-computed tomography to analyze the root canal morphology of maxillary third molars in a Polish population. Root canal morphology of mandibular third molars of an Indian population was also investigated using the clearing technique^32^.

### Maxillary third molar

The internal anatomy of maxillary third molars is particularly complex, whereby three roots are commonly fused, exhibiting a wide variation in canal number and type^8^.

It has been reported that 15% of the studied maxillary molars had only one root, 32% two, 45% three, and 7% four roots^7^. The number of canals ranged from 1 to 6 in single-rooted teeth, 3 to 5 in double-rooted, 2 to 5 and 4 to 5 in teeth with three and four roots respectively. Ng and colleagues^14^ studied the maxillary molars in Burmese population and found that only a quarter of the maxillary third molars had three roots, the rest had a single/fused roots.

A study of Thai maxillary molars found that only around 51% of the maxillary third molars had three separate roots; while the other half had fused or conical roots^5^. The prevalence of mesio-buccal roots with two canals was 28.6% (type II and IV) and the majority of the distal and palatal roots had type I.

In the present study, the prevalence of maxillary third molars of a Jordanian population with three roots was slightly lower than that of Thai^5^, and higher than that of Burmese, American, Turkish and Polish population^6,7,14,26^. Also the prevalence of the teeth with one and two roots was lower than that of Burmese, American, Turkish and Polish population^6,7,14,26^, but higher than that of Thai population^5^. The difference in the prevalence of root numbers may be due to the different ethnic backgrounds or discrepancy in the sample size (Table 5).

**Table 5:** Percentage of root number of maxillary third molar in previous studies and the present one.

Study	Nationality	Sample size	1-root (%)	2-roots (%)	3-roots (%)	4-roots (%)	5-roots (%)
Sidow et al.^7^	American	300	15	32	45	7	
Ng et al.^14^	Burmese	72	19.40	19.40	30.60	5.60	
Alavi et al.^5^	Thai	268	1.30	7	87.70	4	
Sert et al.^6^	Turkish	290	35.50	28.60	34.10	1.70	
Tomaszewska et al.^2^	Polish	78	38.50	–	61.50	–	
Ahmad et al.^16^	Jordanian	89	13.50	5.60	74.20	6.70	
Present study	Jordanian	592	10.80	9.80	70	9.10	0.34

The prevalence of teeth with four roots was higher than that of the aforementioned populations, and in-line with the previous study on a Jordanian population^16^. Maxillary third molar with five roots were recorded in this study for the first time with a prevalence of (0.34%).

The prevalence of canal numbers in this study was consistent with other studies^5,7,14,26^. Moreover, the findings were similar to those of the previous study of a Jordanian population. The number of canals in maxillary third molars in the present study were found to be 3, 4, 2, 1 and 5 in 52.3%, 28.2%,11.4%, 5.9%, and 2% of teeth respectively; compared to 55.1%, 27%, 6.7%, 9% and 2.2% respectively^16^.

In teeth with 3 separate roots; MB root possessed two canals in 36.6% of cases and this was consistent with the findings of Pineda and Kuttler^13^ (31%), Ng et al.^14^, (38.9%), and Alavi et al.^5^, (28.6%), yet higher than that of Pecora and coworkers^33^ (14%). All the DB and the P roots had one canal (type I) which was consistent with other studies^5,14,34^.

### Mandibular third molars

Being the last tooth in the dental arch, mandibular third molar teeth have been associated with significant variation in root patterns and canal systems^8,13^. Generally, two roots and two canals are evident, but infrequently containing three or more canals^35^.

One study provided a morphological description of third molars in the American population^7^. It was reported that 17% of mandibular third molars displayed one root, while the majority (77%) had two roots. The number of canals ranged from 1 to 3, 2 to 6, 3 to 5, and 4 to 5 in teeth with one, two, three and four roots respectively. Another study of Burmese mandibular molars^20^, reported that the prevalence of one, two, three or four canals in mandibular third molars was 3.7%, 55.6%, 37% and 3.7%, respectively. A further study on Thai mandibular molars, reported that of mandibular third molars 68% had two separate roots, 20% had fused roots and 11% had single C-shaped root^36^.

In the present study, mandibular third molar of a Jordanian population was associated with various root and canal morphology. The prevalence of teeth with two, three and four roots was consistent with that of Burmese and Thai population^20,37^, while the prevalence of teeth with a single root was less than that of Thai, American, Turkish, Iranian, and Indian populations^6,7,31,32,36^. Interestingly, the prevalence of single and 4-rooted mandibular third molars in the current study was significantly less than that of a previous study on a similar Jordanian population (Table 6). This could be attributed to the big difference in sample size, whereby 639 mandibular third molars were examined in the current study, compared to only 70 in the previous study on a Jordanian population^16^.

**Table 6:** Percentage of root number of mandibular third molars in previous studies and the present one.

Study	Nationality	Sample size	1-root (%)	2-roots (%)	3-roots (%)	4-roots (%)
Sidow et al.^7^	American	150	17	77	5	1
Gulabivala et al.^20^	Burmese	58	–	100	–	–
Gulabivala et al.^36^	Thai	173	12	86.80	1	1
Sert et al.^6^	Turkish	370	24.80	69.20	5.40	0.27
Kuzekanani et al.^31^	Iranian	150	21	73	5.50	0.50
Ahmad et al.^16^	Jordanian	70	12.90	74.30	8.70	4.30
Singh et al.^32^	Indian	100	15	63	18	4
Present study	Jordanian	639	2.50	89.70	7.30	0.47

The prevalence of three canals (55.7%) in the present study was higher than that of Burmese and Thai population, while the prevalence of two canals (29.4%) was much lower than that of Burmese (55.6%) and Thai (61%) populations^20,36^. Our findings were different from other studies^13,20,36^ where we found much higher incidence of two canals in the mesial root of the teeth with two roots. Most of the distal root had type I canal configuration which was consistent with that of Burmese and Thai populations^20,36^. Again, the difference may be due to the discrepancy in the sample size or the ethnic group.

Lateral canals were found most often in the apical third of the canals. This finding was consistent with the finding of previous studies^20,37^. The prevalence of C-shaped canal was consistent with that found in the American population^7^.

The C-shaped root in the present study showed wide variation of canal numbers and types. The prevalence of C-shaped roots in mandibular third molars was less than that of Thai population^36^. However, C-shaped roots were in-line with other studies on maxillary and mandibular third molars ranging from 3 to 4% of the sample size^7,16,31^.

## Conclusion

Overall, findings of the present study were comparable with other studies. The majority (69.9%) of maxillary third molars had 3 roots, while the majority (89.7%) of mandibular third molars had 2 roots. Nearly half (52.3%) of maxillary third molars and mandibular (55.7%) third molars had three canals. A fifth canal was found in 2% of maxillary third molars. C-shaped canals also occurred. Around 12% of maxillary, and 10% of mandibular third molars displayed lateral canals at the apical third and inter-canal communications. Root canal configuration of third molars vary greatly, and it is crucial for a clinician to be aware of these in case they are undiagnosed radiographically.

## Data Availability

The data sets generated during and/or analyzed during the current study are available from the corresponding author on reasonable request.
